# Experimental signatures of interstitial electron density in transparent dense sodium

**DOI:** 10.1038/s43246-025-00925-w

**Published:** 2025-09-01

**Authors:** Christian V. Storm, Stefano Racioppi, Matthew J. Duff, James D. McHardy, Eva Zurek, Malcolm I. McMahon

**Affiliations:** 1 SUPA, School of Physics and Astronomy, and Centre for Science at Extreme Conditions, The University of Edinburgh, Edinburgh, UK; 2 Department of Chemistry, State University of New York at Buffalo, Buffalo, NY USA

**Keywords:** Structure of solids and liquids, Phase transitions and critical phenomena

## Abstract

The transparent *hP*4 phase of dense sodium (Na), stable above 200 GPa, has been computed to be an electride in which valence electrons are localised on interstitial lattice sites within the structure. However, there is no experimental evidence for this interstitial electron localisation in Na, or indeed in other high-density electride phases. Using static compression and single-crystal X-ray diffraction techniques, we have grown and studied a single-crystal sample of Na in the *hP*4 phase at 223 GPa. Using atomic form factors for *hP*4-Na derived from quantum crystallography techniques, we present experimental results to support the electride nature of this phase.

## Abstract (web summary)

The electride nature of dense sodium in the transparent *hP*4 phase, where valence electrons are interstitially localised, lacks experimental confirmation. Here, the authors use single-crystal X-ray diffraction and quantum crystallographic atomic form factors to provide experimental indication of interstitial electron localisation in *hP*4-Na at 223 GPa, advancing our understanding of high density electrides.

## Introduction

Upon compression, the alkali metals undergo a striking departure from the simple, nearly-free-electron behaviour they exhibit at ambient conditions. Pioneering computational studies of Li^1^ predicted that at high densities this simple metal would transition to an insulating phase with paired atoms, accompanied by the localisation of valence electrons into interstitial lattice sites.

Experimental studies of Li, Na, and heavier alkali metals subsequently found transitions to semiconducting^2^, insulating^3^ and superconducting forms^4,5^, and to a myriad of complex crystal structures^6–8^, including incommensurate phases^6,9,10^. Further calculations have predicted that such behaviour is almost ubiquitous in high density metals^11–13^.

**Fig. 1:**
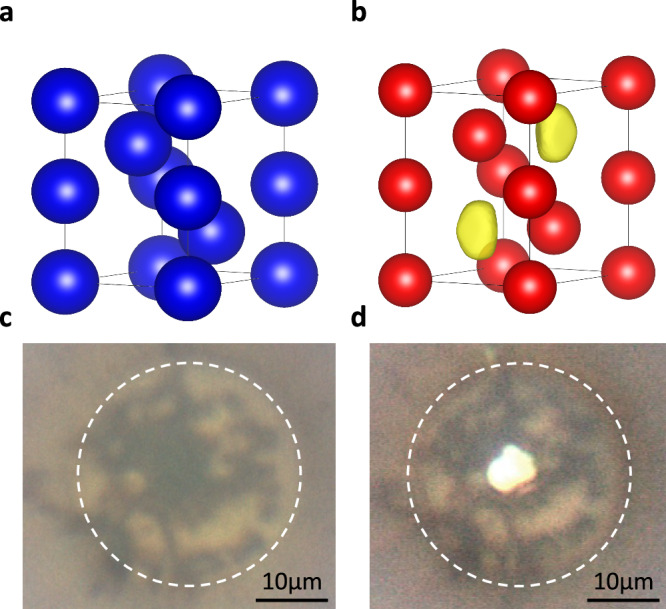
*hP*4-Na structure and single-crystal sample. **a** The *hP*4-Na structure with neutral Na atoms (blue) on the 2*a* and 2*d* sites at (0,0,0) and ($\frac{2}{3}$,$\frac{1}{3}$,$\frac{1}{4}$), respectively. **b** Electride *hP*4-Na with Na ions (red) on the 2*a* and 2*d* sites and isosurfaces of the ELF (isovalue = 0.9) on the 2*c* site at ($\frac{1}{3}$,$\frac{1}{3}$,$\frac{1}{4}$), containing the interstitial charge maxima characteristic of electride states. **c** Image of the transparent single-crystal *hP*4-Na sample at 223 GPa, taken under reflected illumination. **d** Image of the same sample, taken under combined transmitted and reflected illumination. The sample diameter is ~ 5 μm while the diamond anvil culet, outlined with the dashed white circles, has a diameter of 30 μm.

Perhaps the most dramatic manifestation of pressure-induced complexity in any metal is seen in Na. Above 100 GPa, or ~4-fold volume compression, Na exhibits: a dramatic minimum in its melting temperature such that at 110 GPa it is lower than that seen at ambient pressure^14^; several complex crystal structures with as many as 512 atoms per unit cell^15^; an incommensurate host-guest composite structure^10^; and, perhaps most striking of all, an optically transparent, insulating phase above 200 GPa and ~5-fold volume compression^3^. This phase is reported to have a double-hexagonal close-packed(*dhcp*, Pearson symbol *hP*4, Fig. 1a) structure and a 1.3 eV band gap at 200 GPa^3^. At low temperatures, *hP*4 is calculated to be the stable form of Na until it is predicted to become metallic again above 15 TPa^16^, a pressure more than twice that at the centre of Jupiter. At the pressures and temperatures accessible using dynamic compression techniques (250–350 GPa and 700–1900 K), other Na phases are predicted to be more stable than *hP*4-Na^17^.

As initially predicted by Neaton and Ashcroft^18^, the electronic structure of dense insulating forms of Na is characterised by repulsion of the valence 3*s* electron state from the core, which triggers a *s* → *pd* transition^3^. Recent quantum mechanical calculations revealed that the *p* and *d* states hybridise^19^, allowing them to point towards the centre of the lattice interstices where they overlap. This results in the accumulation of charge density at the centre of the interstitial cavities, as evidenced by plots of the electron localisation function (ELF) (Fig. 1b)^19^. Moreover, this charge accumulation is characterised by the presence of a non-nuclear maximum at its centre, which is one of the main topological features of the electron density that can be used to identify an electride state in molecules and molecular crystals^20–22^. For this reason, the insulating *hP*4 phase of Na has been termed an electride by analogy^3^, while a hypothetical high-temperature semi-metallic phase fulfills the criteria of a topological electride^17^. However, neither of these predictions has been experimentally confirmed.

Calculations also predict *hP*4-Na to have very unusual reflectivity—being transparent in one direction but reflecting in the other^23^. Indeed, the authors suggested that this asymmetry would provide a key test to prove that transparent Na does indeed crystallise in the *hP*4-Na structure.

Experimental evidence that dense transparent Na adopts the *hP*4 structure, and the existence of electride-like charge accumulation, can also be provided by X-ray diffraction, which gives a measurement of the electron density within the structure. However, the only X-ray data collected from *hP*4-Na to date observed only three non-overlapped powder diffraction peaks, which had positions, but not intensities, consistent with the *hP*4 structure^3^. To show that transparent Na does indeed assume the *hP*4 structure, and that this phase exhibits electron density accumulation in the interstitial sites, single-crystal rather than powder methods are needed. We have thus exploited Na’s propensity to recrystallise at megabar pressures^10,24^ to perform X-ray single-crystal synchrotron diffraction studies of *hP*4-Na at 223 GPa at room temperature, extended to 310 GPa using X-ray powder diffraction.

We show that transparent Na does indeed adopt the *hP*4 structure above 200 GPa, provide experimental results indicative of electron density accumulation in the interstitial cavities, and show how electride structures can be modelled using quantum crystallography techniques^25,26^, by fitting the experimental X-ray data using atomic form factors derived from density functional theory (DFT) calculations of the charge density.

## Results

Upon compression, the known phase transitions in Na were observed in good agreement with previous investigations^15,24,27^. The Na samples annealed into quasi-single crystals at the *oP*8 → *tI*19 phase transition at 133 GPa, and these crystals were retained on very slow pressure increase into the transparent *hP*4 phase above 206 GPa, see Fig. 1c, d. Visual analysis of the single-crystal samples showed no evidence of the anisotropic reflectivity predicted for *hP*4-Na^23^.

**Fig. 2:**
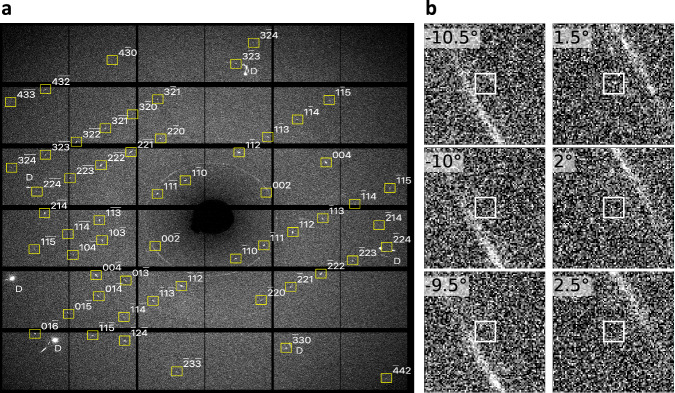
Single-crystal diffraction from *hP*4-Na. **a** Composite single-crystal diffraction image from *hP*4-Na at 223 GPa, created from 120 images collected in 0.5°-steps over a  ± 30° angular scan range. Yellow squares outline the *hP*4-Na reflections with the given *hkl* indices. Reflections from the diamond anvils are marked with the letter *D*, while a number of weak Debye-Scherrer rings arise from the W gasket. The axis of oscillation is vertical. **b** Enlarged views of three consecutive ± 0.5° oscillation images, showing the calculated position of the $(00\bar{2})$ (left) and (002) (right) diffraction peaks from *hP*4-Na, marked by unfilled squares. The arc of intensity is the (110) Debye-Scherrer ring from the W gasket.

A composite rotation image from one single-crystal sample at 223 GPa is shown in Fig. 2a. The sharpness and abundance of the single-crystal reflections meant that determining the structure was straightforward: all observed reflections could be indexed on a hexagonal lattice with *a* = 2.9028(5)Å and *c* = 4.1386(3)Å, and peak intensities consistent with the *hP*4 structure, confirming this as the structure of transparent dense Na. After averaging equivalent reflections, 18 independent peaks remained (see details in Methods section).

In the non-electride *hP*4 structure (Fig. 1a), hereafter called atomic-*hP*4, which has only four neutral Na atoms in the unit cell, the (002) reflection has zero intensity as a result of exact cancellation of the scattering from the Na atoms. However, in the electride-*hP*4 structure, with charge accumulated in the interstitial 2*c* sites (see Fig. 1b), this exact cancellation is broken, and the intensity of the (002) reflection becomes non-zero for sufficiently localised (concentrated) electrons. The presence and intensity of this single peak would thus provide insight into the electron density accumulation. Fig. 2b shows the calculated location of the (002) and equivalent $(00\bar{2})$ reflections. It is clear that both reflections are extremely weak with barely any observable scattering intensity in the 2D diffraction images. Integration of the single-crystal data nonetheless yields an intensity-to-uncertainty ratio (*I*/*σ*_*I*_) of 1.4 for the averaged (002) and $(00\bar{2})$ peaks (see tabulated data in Supplementary Table S3). However, charge localisation also changes the intensity of other low-angle reflections, with the intensity change dependent on the atomic form factor of the Na-ion. This provides a way to confirm the electride-like nature of *hP*4-Na, by comparing the agreement between the observed and calculated intensities using different atomic form factors for the Na-ions and the localised electrons.

Previous attempts to calculate the effects of the localised electrons on the intensity of diffraction peaks have modelled the interstitial electron density by placing an anion at its centre, with the remaining ions being treated as cations. A first estimate of the intensity expected for the (002) peak, and the effects of the interstitial charge accumulation on other peak intensities, can then be made using standard atomic form factors^28^ by placing Na^+^ cations on the 2*a* and 2*d* sites, and H^−^ anions on the 2*c* interstitial sites, thereby simulating a charge accumulation of two electrons at these sites while keeping the total number of electrons per unit cell fixed at 44, the same as for atomic-*hP*4. We will refer to this as the pseudo-*hP*4 model.

The pseudo-*hP*4 model calculates the intensity of the (002) Bragg peak to be non-zero, as expected, but to be only 0.4% of that of the strongest (102) peak. In addition, intensities of the low-angle ($\bar{1}10$) and ($1\bar{1}\bar{1}$) peaks are calculated to decrease by 3% and 11%, respectively, relative to the (102) peak, while intensity changes in the other, higher angle, peaks are much smaller as a result of the rapid fall-off of the atomic form factor of the H^−^ ion with scattering angle (see Supplementary Fig. S2b).

However, it is clear that the interstitial electron density accumulation affects the intensities of *all* the low-angle Bragg peaks, and thus the electride nature of *hP*4-Na can be extracted from the X-ray intensities. Refinement of the single-crystal X-ray data using both the atomic-*hP*4 and pseudo-*hP*4 structures revealed the latter to have a better goodness of fit (GoF) and *R*_1_-factor (3.40 and 10.3%) than the former (3.42 and 10.5%), indicating that the substitution of Na^+^ cations in lieu of Na atoms, and the addition of localised electron density on the interstitial 2*c* sites, provides a better structural model for the *hP*4 phase (see definitions for GoF and *R*_1_ in the Supplementary Information S1.3).

A significantly better fit was obtained using a structure (hereafter called electride-*hP*4) that utilised non-standard atomic form factors for the interstitial charge accumulation and the Na^δ+^ ions derived from DFT calculations of the *hP*4 structure^19^, (see Supplementary Methods S1.1). From the electron density determined from such calculations, one can calculate the structure factors of individual Bragg reflections. A least-squares fit to these calculated structure factors, allowing the atomic form factors of both the Na ions and the interstitial charge accumulation to vary, while constraining the total number of electrons in the unit cell to 44, gave the form factors shown in Supplementary Fig. S2. The calculated form factor for the interstitial charge accumulation shows it contains 1.15 electrons at 223 GPa, in excellent agreement with the value of 1.10 determined by our DFT calculations performed at 190 GPa^19^. The small difference likely arises from the electron charge density in the interstitial sites increasing slightly with pressure.

The form factor for the region wherein the interstitial charge was accumulated is highly peaked in *q*-space, even more so than that of an H atom or a H^−^ ion (Supplementary Fig. S2b), indicating a highly diffuse electron density in real space. As a result, the presence of the accumulated electron density contributes only to the lowest-angle reflections, and the intensity of the (002) reflection, despite having the 3^rd^ lowest Bragg angle, is calculated to be extremely weak. The electride-*hP*4 model gave by far the best fit to the X-ray data, with a GoF and *R*_1_-factor of 3.18 and 9.7%, respectively.

**Fig. 3:**
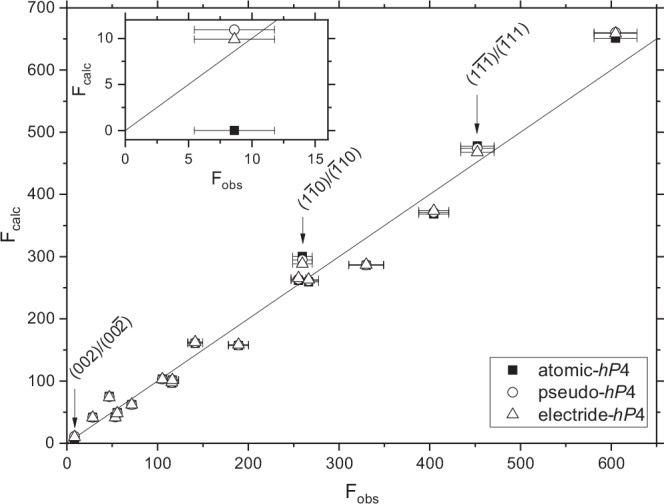
Comparison of the fits of the atomic-*hP*4, pseudo-*hP*4, and electride-*hP*4 structural models. The structure factors measured in the experiment (F_obs_) are plotted against those calculated by the three models (F_calc_). In a perfect fit, all data points would lie on the solid line depicting F_calc_ = F_obs_. The arrows mark the locations of the three reflections most affected by the change of model, and the inset shows an enlarged view of the very weak $(002)/(00\bar{2})$ reflection, which is calculated to be non-zero only in the pseudo-*hP*4 and electride-*hP*4 models. The structure factors have been multiplied by fitted scale factors, and the error bars on F_obs_ are propagated from uncertainties on the observed intensities.

These results are summarised in Table 1, and the improved fit is illustrated in Fig. 3, where the structure factors calculated from the experimentally observed reflection intensities (F_obs_) are plotted against the structure factors calculated from the three different structure models (F_calc_). The improved fits provided by the two models that account for the electron density accumulation are reflected in a better overall F_obs_–F_calc_ agreement, with the improved fits being particularly evident for the three lowest-angle reflections - the (002), $(1\bar{1}0)$, and $(1\bar{1}\bar{1})$ - where the effects of the charge accumulation are largest. In each case, the DFT-derived electride-*hP*4 model gives the best agreement with the observed peak intensities (see Supplementary Table S3).

**Table 1:** The three *hP*4-Na structural models used to fit the single-crystal X-ray diffraction data obtained at 223 GPa, and the values of the goodness of fit (GoF), *R*_1_, and Akaike Information Criterion (AIC) for each

Model	Form Factors	Wyckoff Sites	GoF	*R*_1_ (%)	AIC
Atomic-*hP*4	Na^28^	2*a*, 2*d*	3.42	10.5	293
Pseudo-*hP*4	Na^+^^28^	2*a*, 2*d*	3.40	10.3	281
	H^−^^28^	2*c*			
Electride-*hP*4	Na^δ+^ (DFT)	2*a*, 2*d*	3.18	9.7	269
	Charge Accumulation (DFT)	2*c*			

The atomic- and electride- models have only two variable parameters while the pseudo-*hP*4 has three (see Supplementary Table S5).

To further quantify the improvement in the fit between these models, we have used the Akaike Information Criterion (AIC)^29^, previously used to distinguish between models of host-guest complexes with varying stoichiometries^30^, and note a significant improvement of ΔAIC = 12 for the pseudo-*hP*4 model and 24 for the electride-model, relative to the atomic-*hP*4 model (see Table 1). A ΔAIC of >10 generally indicates that there is no support for the higher-AIC model, which are the atomic- and pseudo-*hP*4 models in this case^31^. Note that while we have included the extremely weak $(002)/(00\bar{2})$ reflections in our analysis throughout, their omission results in ΔAIC = 23 for the electride model relative to the atomic models, and therefore does not affect our conclusions.

**Fig. 4:**
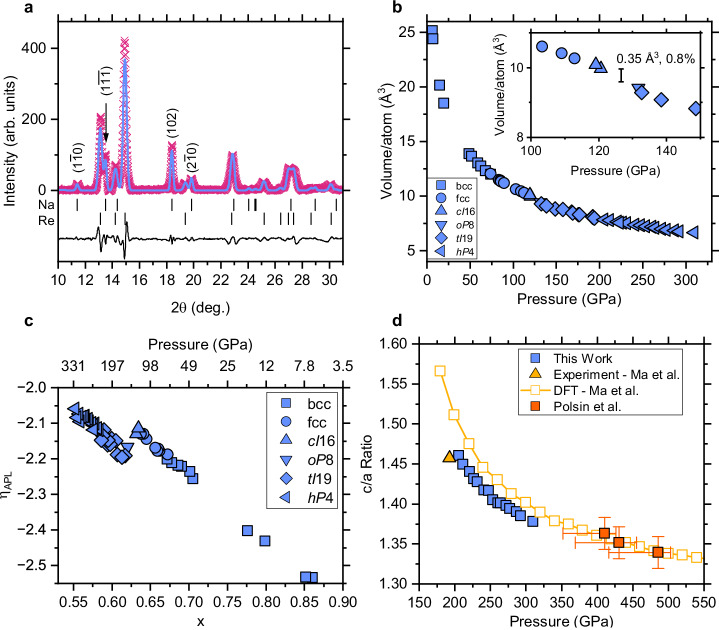
Pressure-dependence of *hP*4-Na phase to 310 GPa. **a** A two-phase *hP*4-Na/*hcp*-Re Le Bail fit (blue solid line) to the background-subtracted diffraction profile (magenta crosses) from Na at 310 GPa. Tick marks beneath the profile show the calculated peak positions of both phases, and the solid black line is the difference between the data and the Le Bail fit. **b** Compressibility of Na to 310 GPa showing the different phases and the volume change of 0.8% (Δ*V*/*V*_0_) observed at the *cI*16 → *oP*8 transition at 123 GPa. **c**, The linearised compressibility data for Na up to 310 GPa, highlighting the strongly linear compressibility exhibited over the entire pressure range. **d** The measured *c*/*a* axial ratio of *hP*4-Na up to 310 GPa (blue squares), with previous experimental (yellow triangle) and computational (yellow squares) results from Ma et al.^3^, and data from ramp-compressed samples by Polsin et al. (orange squares)^43^.

To investigate the behaviour of Na to higher pressures, we compressed one of the samples until anvil failure at 310 GPa. The highest-pressure diffraction profile, and a Le Bail fit to it, are shown in Fig. 4a. The quality of the fit is excellent, and the *hP*4-Na lattice parameters at 310 GPa are *a* = 2.8132(6)Å and *c* = 3.8759(11)Å, giving an atomic volume of 6.641(3)Å^3^ (*V*/*V*_0_ = 0.168), and hence a volume compression ratio of 5.95, with *V*_0_ taken as 39.499 Å^3^/atom^32^.

The compression curve (Fig. 4b) illustrates the high compressibility of Na, which exhibits a ~3-fold volume compression ratio at only 50 GPa. As the aim of our study was to compress Na to multi-megabar pressures, we spent little effort on studying the well-known lower-pressure phases, and collected only a few diffraction profiles below 50 GPa. The inset to Fig. 4a shows the volume discontinuity of 0.35 Å^3^/atom at the *cI*16-*oP*8 transition, in excellent agreement with the previously reported 0.33 Å^3^/atom^15^, and constituting a ~0.8% reduction in *V*/*V*_0_.

To further investigate any changes or anomalies in the compressibility of Na, we plotted the data in linearised space with respect to the Adapted Polynomial of order L (APL) equation of state^33,34^ (see Supplementary Methods S1.2 and refs. ^35,36^ for details). Fig. 4c shows the remarkably linear behaviour of Na, in contrast to the highly non-linear behaviour exhibited by other alkali metals, such as Rb^37^. The volume discontinuity at the *cI*16 → *oP*8 transition is obvious at *x* = 0.63, but in the phases both below and above this transition, the data show strong linearity with very similar slopes. Despite undergoing five phase transitions, exhibiting great structural complexity, and being compressed to six times its initial density, the compressibility of Na up to 300 GPa is that of a “regular” metal^38^.

As calculated by Ma et al.^3^, the *c*/*a* axial ratio of *hP*4-Na decreases with pressure (Fig. 4d), though we measure this ratio to be systematically 0.2–0.3 below Ma’s computational results. Our data are, however, in good agreement with the *c*/*a* ratio *measured* by Ma et al. at 190 GPa. The dynamic-compression data from Polsin et al.^39^ are also shown, but are difficult to compare to our results due to the non-overlapping pressure domains and significant uncertainties.

In summary, we have grown a single-crystal sample of transparent dense Na, stable above 200 GPa, and have collected single-crystal X-ray diffraction data from this phase. We have confirmed that it has the hexagonal *hP*4 structure, but that a structural model containing neutral Na atoms is insufficient to fully describe the observed Bragg intensities. We have therefore developed a structural model using optimised atomic form factors for the Na cations and interstitial electron density, as determined from charge densities derived from DFT calculations and quantum crystallographic techniques, and have shown that this electride model^19^ provides the best fit to our diffraction data. Each interstitial lattice site contains 1.15 electrons, and the electron density is more extended in real space than that of an H-atom or H^−^ anion. We have shown that *hP*4-Na is stable up to at least 310 GPa at 298 K, where its density is increased six-fold. Finally, visual analysis of the *hP*4 single crystal showed no evidence of the anisotropic reflectivity predicted previously, but further quantitative studies of both the reflectivity and refractive index of dense transparent Na are planned.

## Methods

### Experimental method

Sodium metal of 99.95% purity supplied by Sigma-Aldrich was loaded into Boehler-Almax plate diamond anvil cells (DACs)^40^ in a dry argon atmosphere (O_2_ < 0.1 ppm, H_2_O < 0.1 ppm). Three DACs were prepared, two of which were loaded without an in-situ pressure marker, with the pressure instead determined from the W and Re gasket diffraction peaks, respectively, and their respective equations of state^41,42^. The third was loaded with a Cu sphere as a pressure marker, and had a Re gasket, with pressure estimated from the Cu equation of state^43^. Angle-dispersive X-ray diffraction studies were carried out on beamline P02.2 at the Petra-III synchrotron (Germany), and on beamline ID27 at the ESRF-EBS synchrotron (France). At the former, a monochromatic X-ray beam of wavelength *λ* = 0.4845Å, focused down to a FWHM of 0.85 × 0.85 μm, was used, and the diffraction data were recorded on a Perkin-Elmer XRD 1621 detector, placed ~330 mm from the sample. Polycrystalline CeO_2_ and single-crystal enstatite were used to calibrate the exact sample-detector distance and detector tilts. At the ESRF-EBS, a monochromatic X-ray beam of wavelength *λ* = 0.3738 Å, focused down to a FWHM of 0.5 × 0.5 μm, was used, and the diffraction data were recorded on an EIGER2 X CdTe 9M detector, placed ~ 200 mm from the sample.

Data from the *hP*4 single crystals were collected at both synchrotrons in a sequence of contiguous 0.5° oscillations over the full angular ranges permitted by the cell. The exposure time was 1 s per frame.

The intensities of all accessible reflections were integrated using Crysalis Pro^44^ and were corrected for changes in the illuminated sample volume due to the sample rotation and absorption by the diamond anvils. After rejecting reflections with bad peak profiles (*I*/*σ*_*I*_ > 10 and profile agreement < 0.8), 41 reflections remained, and analysis of the systematic absences showed them to be consistent with the space group P6_3_/mmc, with an internal intensity agreement *R*_*int*_ of 4.7%. Filtering out weak reflections with *I* < 3*σ*_*I*_ left 28 reflections; however, this also excluded the (002) reflection from the analysis. Since this reflection was expected to be very weak but was of particular interest to the study, we re-included this reflection, yielding a total of 18 independent reflections after averaging. We plot the propagated uncertainties in Fig. 3.

Le Bail and Rietveld refinements were performed in Jana2006^45^, and DIOPTAS^46^ was used for calibration, integration, and general XRD data analysis. Unit cell parameters for all phases of Na, as well as those of W, Re, and Cu, were obtained from Le Bail profile fits or analysis of the single-crystal data.

In the *hP*4 structure, the atomic coordinates of the Na ions and the localised charge accumulation are fixed by symmetry, and the refinable parameters were thus an overall scale factor, and atomic displacement parameters (ADPs) *U*_*iso*_ for the atoms and ions. In all structural models, the ADPs of two independent Na atoms or ions were constrained to be the same. When fitting the electride-*hP*4 model, the *U*_*iso*_ for the charge accumulation refined to zero within error, and was therefore fixed to zero.

### Theoretical methods

In order to obtain the form factors of the Na ions and the non-nuclear maxima (NNM) in the electride phase of Na*-hP*4, we calculated the static structure factors (*F*_*hkl*_) of the 465 Bragg reflections with *d* > 0.2 Å. The structure factors were obtained from the electron density of Na*-hP*4 calculated with DFT and using an atomic orbitals basis set with the code Crystal17^47^. The calculation was performed using the geometry of Na-*hP*4 at 223 GPa, measured in this work. The screened hybrid exchange-correlation functional HSE06^48–50^ was adopted in combination with a modified quadruple-*ζ* double polarised atom-centred basis-set^51^, which was reported and used in ref. ^19^ (named Na). The *k*-point grid was generated with the Monkhorst-Pack method^52^, using a shrinking factor of 32 along the reciprocal lattice vectors (32 × 32 × 32 grid in *k*-space), while the convergence threshold of the total energy was set to 10^–8^ Ha. The band gap calculated at this level of theory equalled 1.71 eV, which coincides with previous predictions^3^ made at the GW level of theory^53^ using the plane-wave based code VASP^54^, confirming the completeness of our basis set and accuracy of our level of theory.

## Supplementary information

Supplementary Information

## Data Availability

The data for Fig. 4b are presented in Supplementary Tables S1 & S2, respectively. The observed single-crystal intensities, averaged over symmetry-equivalents), are presented in Supplementary Table S3 together with calculated intensities. All other data supporting the findings of this study are available from the corresponding author upon reasonable request. The atomic-*h**P*4 structure is available on the ICSD with the identifier 2325643.

## References

[1] Neaton, J. B. & Ashcroft, N. W. Pairing in dense lithium. *Nature* **400**, 141–144 (1999).

[2] Matsuoka, T. & Shimizu, K. Direct observation of a pressure-induced metal-to-semiconductor transition in lithium. *Nature* **458**, 186–189 (2009). 19279633 10.1038/nature07827

[3] Ma, Y. et al. Transparent dense sodium. *Nature* **458**, 182–185 (2009). 19279632 10.1038/nature07786

[4] Shimizu, K., Ishikawa, H., Takao, D., Yagi, T. & Amaya, K. Superconductivity in compressed lithium at 20 K. *Nature* **419**, 597–599 (2002). 12374973 10.1038/nature01098

[5] Struzhkin, V. V., Eremets, M. I., Gan, W., Mao, H. K. & Hemley, R. J. Superconductivity in dense lithium. *Science* **298**, 1213–1215 (2002). 12386338 10.1126/science.1078535

[6] McMahon, M. I., Rekhi, S. & Nelmes, R. J. Pressure dependent incommensuration in Rb-IV. *Phys. Rev. Lett.* **87**, 055501 (2001). 11497781 10.1103/PhysRevLett.87.055501

[7] Nelmes, R. J., McMahon, M. I., Loveday, J. S. & Rekhi, S. Structure of Rb-III: novel modulated stacking structures in alkali metals. *Phys. Rev. Lett.* **88**, 155503 (2002). 11955205 10.1103/PhysRevLett.88.155503

[8] Marqués, M. et al. Crystal structures of dense lithium: A metal-semiconductor-metal transition. *Phys. Rev. Lett.* **106**, 095502 (2011). 21405633 10.1103/PhysRevLett.106.095502

[9] McMahon, M. I., Nelmes, R. J., Schwarz, U. & Syassen, K. Composite incommensurate K-III and a commensurate form: study of a high-pressure phase of potassium. *Phys. Rev. B* **74**, 140102 (2006).

[10] Lundegaard, L. F. et al. Single-crystal studies of incommensurate Na to 1.5 Mbar. *Phys. Rev. B* **79**, 064105 (2009).

[11] McMahon, M. I. & Nelmes, R. J. High-pressure structures and phase transformations in elemental metals. *Chem. Soc. Rev.* **35**, 943 (2006). 17003900 10.1039/b517777b

[12] Pickard, C. J. & Needs, R. J. Aluminium at terapascal pressures. *Nat. Mater.* **9**, 624–627 (2010). 20622863 10.1038/nmat2796

[13] Miao, M. S. & Hoffmann, R. High pressure electrides: a predictive chemical and physical theory. *Acc. Chem. Res.* **47**, 1311–1317 (2014). 24702165 10.1021/ar4002922

[14] Gregoryanz, E., Degtyareva, O., Somayazulu, M., Hemley, R. J. & Mao, H. K. Melting of dense sodium. *Phys. Rev. Lett.* **94**, 185502 (2005). 15904383 10.1103/PhysRevLett.94.185502

[15] Gregoryanz, E. et al. Structural diversity of sodium. *Science* **320**, 1054–1057 (2008). 18497293 10.1126/science.1155715

[16] Li, Y. et al. Metallic icosahedron phase of sodium at terapascal pressures. *Phys. Rev. Lett.* **114**, 125501 (2015). 25860756 10.1103/PhysRevLett.114.125501

[17] Wang, B., Hilleke, K. P., Wang, X., Polsin, D. N. & Zurek, E. Topological electride phase of sodium at high pressures and temperatures. *Phys. Rev. B* **107**, 184101 (2023).

[18] Neaton, J. B. & Ashcroft, N. W. On the constitution of sodium at higher densities. *Phys. Rev. Lett.* **86**, 2830–2833 (2001). 11290050 10.1103/PhysRevLett.86.2830

[19] Racioppi, S., Storm, C. V., McMahon, M. I. & Zurek, E. On the electride nature of Na-hP4. *Angew. Chem. Int. Ed.* **62**, e202310802 (2023). 10.1002/anie.202310802 37796438

[20] Dye, J. L. Electrides: From 1d Heisenberg chains to 2d pseudo-metals. *Inorg. Chem.* **36**, 3816–3826 (1997).

[21] Postils, V., Garcia-Borrás, M., Solá, M., Luis, J. M. & Matito, E. On the existence and characterization of molecular electrides. *Chem. Commun.* **51**, 4865–4868 (2015). 10.1039/c5cc00215j 25697671

[22] Racioppi, S. & Zurek, E. High-pressure electrides: a quantum chemical perspective. *Annu. Rev. Mater. Res.* **55**, 18 (2025).

[23] Gatti, M., Tokatly, I. V. & Rubio, A. Sodium: a charge-transfer insulator at high pressures. *Phys. Rev. Lett.* **104**, 216404 (2010). 20867123 10.1103/PhysRevLett.104.216404

[24] McMahon, M. I. et al. Structure of sodium above 100 GPa by single-crystal x-ray diffraction. *Proc. Natl Acad. Sci. USA* **104**, 17297–17299 (2007). 17947379 10.1073/pnas.0709309104 PMC2077250

[25] Genoni, A. & Macchi, P. Quantum crystallography in the last decade: developments and outlooks. *Crystals* **10**, 473 (2020).

[26] Racioppi, S. & Zurek, E. Looking at high-pressure electrides through the lens of quantum crystallography: the case of simple cubic calcium. *Acta. Cryst. B*. **81** 256–265 (2025). 10.1107/S2052520625001647 40131789

[27] Hanfland, M., Loa, I. & Syassen, K. Sodium under pressure: BCC to FCC structural transition and pressure-volume relation to 100 GPa. *Phys. Rev. B* **65**, 184109 (2002).

[28] Brown, P. J., Fox, A. G., Maslen, E. N., O’Keefe, M. A. & Willis, B. T. M. Intensity of diffracted intensities. In *International Tables for Crystallography*, 554–595 (International Union of Crystallography, 2006).

[29] Akaike, H. A new look at the statistical model identification. *IEEE Trans. Autom. Control* **19**, 716–723 (1974).

[30] Ikemoto, K., Takahashi, K., Ozawa, T. & Isobe, H. Akaike’s information criterion for stoichiometry inference of supramolecular complexes. *Angew. Chem. Int. Ed.* **62**, e202219059 (2023). 10.1002/anie.202219059 36764927

[31] Burnham, K. & Anderson, D. *Model Selection and Multimodel Inference, 2nd Edn.* (Springer, 2004).

[32] Barrett, C. S. X-ray study of the alkali metals at low temperatures. *Acta Crystallogr.* **9**, 671–677 (1956).

[33] Holzapfel, W. B. Physics of solids under strong compression. *Rep. Prog. Phys.* **59**, 29–90 (1996).

[34] Holzapfel, W. B. Equations of state for solids under strong compression. *High. Press. Res.* **16**, 81–126 (1998).

[35] Finnegan, S. E. et al. High-pressure structural systematics in samarium up to 222 GPa. *Phys. Rev. B* **101**, 174109 (2020).

[36] Finnegan, S. E. et al. High-pressure structural systematics in neodymium up to 302 GPa. *Phys. Rev. B* **103**, 134117 (2021).

[37] Storm, C. V. et al. Behavior of rubidium at over eightfold static compression. *Phys. Rev. B* **103**, 224103 (2021).

[38] Holzapfel, W. B. Equations of state for solids under strong compression. *Z. Fur Kristallographie* **216**, 473–488 (2001).

[39] Polsin, D. N. et al. Structural complexity in ramp-compressed sodium to 480GPa. *Nat. Commun.* **13**, 2534 (2022). 35534461 10.1038/s41467-022-29813-4 PMC9085792

[40] Boehler, R. & De Hantsetters, K. New anvil designs in diamond-cells. *High. Press. Res.* **24**, 391–396 (2004).

[41] Dewaele, A., Loubeyre, P. & Mezouar, M. Equations of state of six metals above 94 GPa. *Phys. Rev. B* **70**, 094112 (2004).

[42] Sakai, T. et al. High pressure generation using double-stage diamond anvil technique: problems and equations of state of rhenium. *High. Press. Res.* **38**, 107–119 (2018).

[43] Fratanduono, D. E. et al. Probing the solid phase of noble metal copper at terapascal conditions. *Phys. Rev. Lett.* **124**, 015701 (2020). 31976690 10.1103/PhysRevLett.124.015701

[44] Rigaku Oxford Diffraction. CrysAlis Pro v171.42.49 https://www.rigaku.com/products/crystallography/crysalis (2022).

[45] Petrícek, V., Dušek, M. & Palatinus, L. Crystallographic computing system JANA2006: general features. *Z. fur Kristallographie* **229**, 345–352 (2014).

[46] Prescher, C. & Prakapenka, V. B. DIOPTAS: a program for reduction of two-dimensional X-ray diffraction data and data exploration. *High. Press. Res.* **35**, 223–230 (2015).

[47] Dovesi, R. et al. Quantum-mechanical condensed matter simulations with crystal. *WIREs Computational Mol. Sci.* **8**, e1360 (2018).

[48] Heyd, J. & Scuseria, G. E. Assessment and validation of a screened coulomb hybrid density functional. *J. Chem. Phys.* **120**, 7274–7280 (2004). 15267636 10.1063/1.1668634

[49] Heyd, J. & Scuseria, G. E. Efficient hybrid density functional calculations in solids: assessment of the Heyd-Scuseria-Ernzerhof screened coulomb hybrid functional. *J. Chem. Phys.* **121**, 1187–1192 (2004). 15260659 10.1063/1.1760074

[50] Heyd, J., Peralta, J. E., Scuseria, G. E. & Martin, R. L. Energy band gaps and lattice parameters evaluated with the heyd-scuseria-ernzerhof screened hybrid functional. *J. Chem. Phys.* **123**, 174101 (2005). 10.1063/1.2085170 16375511

[51] Pritchard, B. P., Altarawy, D., Didier, B., Gibson, T. D. & Windus, T. L. New basis set exchange: An open, up-to-date resource for the molecular sciences community. *J. Chem. Inf. Model.* **59**, 4814–4820 (2019). 31600445 10.1021/acs.jcim.9b00725

[52] Monkhorst, H. J. & Pack, J. D. Special points for Brillouin-zone integrations. *Phys. Rev. B* **13**, 5188–5192 (1976).

[53] Shishkin, M. & Kresse, G. Self-consistent GW calculations for semiconductors and insulators. *Phys. Rev. B* **75**, 235102 (2007). 10.1103/PhysRevLett.99.246403 18233465

[54] Kresse, G. & Furthmüller, J. Efficient iterative schemes for ab initio total-energy calculations using a plane-wave basis set. *Phys. Rev. B* **54**, 11169–11186 (1996). 10.1103/physrevb.54.11169 9984901

