# Supplementary material for: Experimental signatures of interstitial electron density in transparent dense sodium
Source: Commun Mater. 2025 Sep 1;6(1):201. doi: 10.1038/s43246-025-00925-w (PMC12401722; doi:10.1038/s43246-025-00925-w)
Supplement: Supplementary file 1 — Supplementary Information [file 43246_2025_925_MOESM1_ESM.pdf]

Supplementary Information

Experimental Signatures of Interstitial Electron Density in Transparent Dense Sodium

Christian V. Storm[1*], Stefano Racioppi[2], Matthew J. Duff[1],
James D. McHardy[1], Eva Zurek[2] and Malcolm I. McMahon[1]

[1]SUPA, School of Physics and Astronomy, and Centre for Science at Extreme Conditions, The University of Edinburgh, Peter Guthrie Tait Road, Edinburgh, EH9 3FD, United Kingdom.

[2]Department of Chemistry, State University of New York at Buffalo, Buffalo, 14260-3000, NY, USA.

[*]Corresponding author(s). E-mail(s): cstorm@ed.ac.uk

S1 Supplementary Methods

S1.1 Optimised Atomic Form Factors for the Na-ions and Non Nuclear Maxima in Transparent Dense *hP*4 Sodium

In order to calculate the intensities of the diffraction peaks from electride-$hP4$-Na accurately, and provide an optimised fit to the single-crystal data collected from it, it was essential that we obtained the correct atomic form factors $f(q)$ for the Na ions and for the electride-like interstitial charge accumulation (ICA) and the resulting Na ions. The atomic form factor of an atom is the Fourier transform of its electron charge density $\rho(r)$ and for most crystallographic calculations the electron density, and hence the form factors, are assumed to be spherically symmetric and depend only on the scattering vector q. For form factors with q between 0 and 25 Å^{-1}, the q-dependence is found to be well-approximated by a sum of 4 Gaussians:

$$f(q) = \sum_{i=1}^{4} a_i \exp\left(-b_i \left(\frac{q}{4\pi}\right)^2\right) + c \tag{S1}$$

where the values of a_i, b_i and c for neutral atoms and their ions are tabulated [1], and $\Sigma a_i + c$ is equal to the total number of electrons in the atom or ion. For example, the atomic form factor for a neutral Na atom is shown in Fig. S1, along with the 4 Gaussians and constants used to model it in crystallographic calculations [1].

In order to obtain the form factors of the Na ions and the ICA in the electride phase of Na-$hP4$, we calculated, using DFT, the structure factors of the 465 Bragg reflections with $d > 0.2$Å. The electron density of Na-$hP4$, having the geometry measured from the single-crystal diffraction analysis at 223

GPa, was computed with Crystal17 [2] using the HSE06 exchange-correlation functional [3–5] and the modified quadruple-ζ double polarised atom-centred basis-set reported in Ref. [6] (named Na). We then set up a crystallographic calculation of the same structure factors using an $hP4$-Na structure with identical Na ions on the $2a$ and $2d$ Wyckoff sites, and ICA on the $2c$ sites. As a starting model we used the tabulated atomic form factors of Na^+ and H^- ions [1] to model the Na ions and ICA, respectively. The use of H^- ions ensured that the total number of electrons in the unit cell was 44, the same as for 4 neutral Na atoms. We then minimised the difference between the DFT and crystallographic structure factors by allowing the nine values of a_i, b_i and c for both the Na ions and ICA to vary, subject to the single constraint that there were 44 electrons in the unit cell. Those fits in which we refined all nine values of a_i, b_i and c for the ICA were found to be unstable. However, we found that stable refinements, and excellent agreement between the structure factors, could be obtained by refining only a_1 and b_1 for the ICA, thereby modelling its atomic form factor with a single Gaussian, with all structure factors agreeing within ± 0.04, and 95% agreeing within ± 0.01. The best-fitting atomic form factors for the Na ions and ICA are shown in Fig. S2, along with those for neutral Na and H atoms, and H^- anions, for comparison. The crystallographic fit suggests that there are 10.425 electrons in each Na ion and 1.149 electrons in each ICA. The form factor of the Na ion in $hP4$-Na is very similar to that of an Na atom, although with reduced scattering at small-q. The form factor for the ICA drops off rapidly in q, even more so than that of a H^- anion, and hence has an electron density in real space that is more diffuse than that of an H^- anion. An important consequence of this is that there is essentially no scattering from the ICA above q=4 Å^{-1} (Fig. S2b), and hence $2\theta \sim 15°$ in the current study: above that angle the scattering from

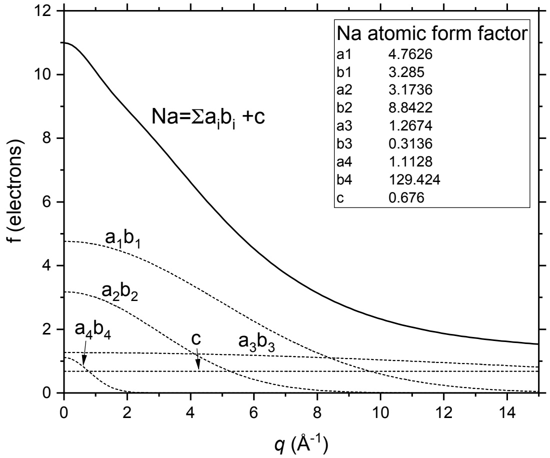

Supplementary Fig. S1. The atomic form factor of a neutral Na atom [1], showing how the curve is parameterised for crystallographic calculations as the sum of four Gaussians and a constant, which are shown as dashed lines.

the electride-$hP4$ structure is indistinguishable from that of the atomic-$hP4$ comprising only 4 neutral Na atoms.

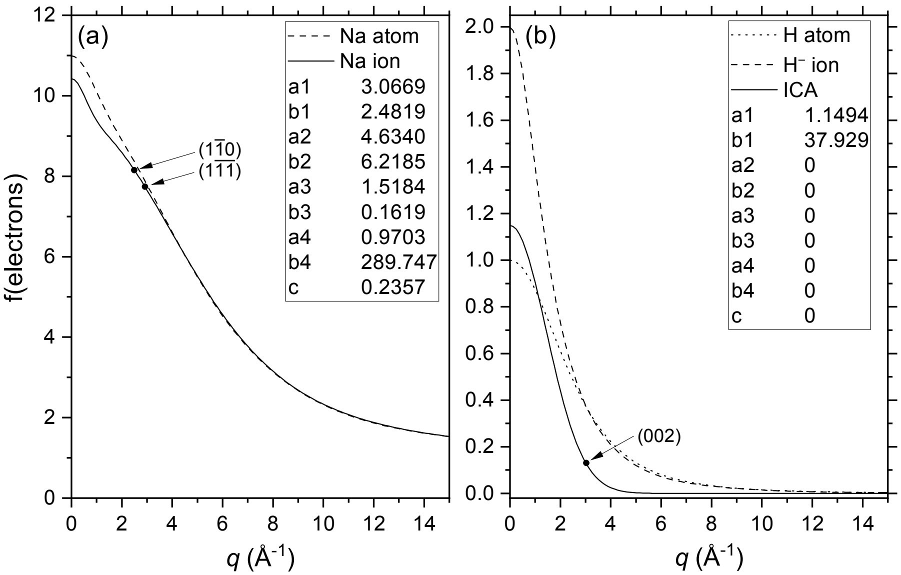

Supplementary Fig. S2. The best fitting atomic form factors for (a) the Na ions and (b) the interstitial charge accumulation (ICA) in the electride-$hP4$ structure, as obtained by fitting to the structure factors obtained from DFT calculations of the charge density [6]. The parameters used to model the form factors are given in the legends. The atomic form factors of (a) an Na atom, and (b) an H atom and H^- anion are shown for comparison. The arrows in panel (a) show the atomic form factor of the Na ions used to calculate the intensities of the low-angle ($1\bar{1}0$) and ($1\bar{1}\bar{1}$) reflections from $hP4$-Na at 223 GPa. These are reduced slightly compared to those of the Na atoms, reducing the calculated intensities of these Bragg peaks in the electride-$hP4$ model (see Table S3). The atomic from factor of the ICA used to calculate the intensity of the (002) reflection in the electride-$hP4$ model is shown in Panel (b) and is only $\sim$0.13 electrons, or 1/3 of an H-atom or H^--anion at the same scattering angle.

S1.2 Linearising the Compression Data

The APL equation of state form is given by [7]:

$$P(x) = 3K_0 \frac{(1-x)}{x^5} e^{c_0(1-x)} \left[1 + x \sum_{k=2}^{L} c_k (1-x)^{k-1} \right] \qquad \text{(S2)}$$

where K_0 is the zero-pressure bulk modulus, K_0' is its pressure derivative, $x = (V/V_0)^{1/3}$, $c_0 = -\ln(3K_0/P_{FG0})$, $c_2 = (3/2)(K_0' - 3) - c_0$, c_k are independent

fitting parameters for $k \in [3..L]$, $P_{FG0} = a_{FG0}(Z/V_0)^{(5/3)}$ is the Fermi-gas pressure, Z is the atomic number, and $a_{FG0} = 2337\,\mathrm{GPa\AA}^5$ is a constant. Note that in the lowest-order AP1 ($L = 1$) case, the summation term is zero and the only fitted parameters are the ambient volume V_0 and bulk modulus K_0, with K_0' being calculated from $K_0' = 3 + (2/3)c_0$.

The corresponding linearised form is given by

$$\eta_{APL}(x) = \ln\left[\frac{Px^5}{P_{FG0}\,(1-x)}\right] \tag{S3}$$

with variables and constants as in Eq. (S2). In this space, "regular" metals exhibit linear or quasi-linear behaviour, see for instance Al and Cu [8], respectively, whereas "irregular" metals such as Rb [9], Sm [10], or Nd [11] are strongly non-linear owing to changes in their compressibility with pressure arising from electronic transitions.

S1.3 Goodness of Fit, R_1, and AIC

R_1 values are calculated from the structures factors and is given by

$$R_1 = \frac{\sum_i \|F_{obs,i} - F_{calc,i}\|}{\sum_i \|F_{obs,i}\|} \tag{S4}$$

and Goodness of Fit (GoF) was calculated as

$$GoF = \sqrt{\frac{\sum w(\|F_{obs}\| - \|F_{calc}\|)^2}{n-p}} \tag{S5}$$

with $w_i = \frac{1}{\sigma^2_{F_{obs}} + 0.0001 F^2_{obs}}$ where n is number of independent reflections, p is the number of refined parameters.

We calculate the Akaike Information Criterion from [12]

$$AIC = 2k - 2\ln L \tag{S6}$$

where k are the degrees of freedom and $\ln L$ is the log-likelihood

$$\ln L = -\frac{1}{2}\sum_{i=1}^{n}\left[\ln(2\pi\sigma^2_{F_{obs,i}}) + \frac{(F_{obs,i} - F_{calc,i})^2}{\sigma^2_{F_{obs,i}}}\right] \tag{S7}$$

S2 Supplementary Tables

Phase	Pressure (GPa)	Volume (Å^3/atom)
bcc	0	39.499 [13]
bcc	6.5	25.152(11)
bcc	7.3	24.387(7)
bcc	15.1	20.134(4)
bcc	19.9	18.494(6)
bcc	49.1	13.857(4)
bcc	52.2	13.632(4)
bcc	59.3	13.011(4)
bcc	62.6	12.736(7)
bcc	68.2	12.319(4)
bcc	73.9	11.961(6)
fcc	73.9	12.027(3)
fcc	83.6	11.457(5)
fcc	85.2	11.392(4)
fcc	86.0	11.320(3)
fcc	89.5	11.166(2)
fcc	103.3	10.601(3)
fcc	109.1	10.4136(14)
fcc	112.9	10.260(4)
$cI16$	119.2	10.0851(5)
$cI16$	120.7	9.962(3)
$oP8$	131.7	9.423(4)
$tI19$	132.6	9.289(15)
$tI19$	138.5	9.0849(13)
$tI19$	148.7	8.82(3)
$tI19$	157.4	8.7542(15)
$tI19$	168.0	8.52(3)
$tI19$	169.2	8.427(2)
$tI19$	175.3	8.28(3)
$tI19$	176.5	8.310(2)
$tI19$	191.9	8.10(3)
$tI19$	191.9	8.038(2)
$tI19$	194.2	7.9468(13)
$hP4$	205.5	7.841(2)
$hP4$	211.7	7.755(3)
$hP4$	221.0	7.642(3)
$hP4$	223.0	7.550(3)
$hP4$	226.5	7.543(3)
$hP4$	231.7	7.479(3)
$hP4$	241.1	7.357(4)
$hP4$	247.6	7.291(3)
$hP4$	253.7	7.196(3)
$hP4$	260.6	7.145(3)
$hP4$	264.5	7.089(3)
$hP4$	272.2	6.984(4)
$hP4$	277.3	6.882(5)
$hP4$	286.8	6.845(3)
$hP4$	292.6	6.744(4)
$hP4$	309.6	6.641(3)

Supplementary Table S1. Compressibility Data. The pressure-dependence of the atomic volume of Na to 310 GPa. For the atomic volume at ambient pressure we use the value 39.499 Å^3 from Barrett [13]. For the incommensurate $tI19$ phase, there were insufficient diffraction peaks from the guest component of the structure to determine the incommensurate ratio γ, which is required to determine the number of atoms per unit cell. We thus used a fixed value of 19.3 atoms per unit cell, as determined previously at 147 GPa [14].

Pressure (GPa)	c/a-Ratio
205.5	1.4602(3)
211.7	1.4492(3)
221.0	1.4406(4)
226.5	1.4312(4)
231.7	1.4275(4)
241.1	1.4172(5)
247.6	1.4164(3)
253.7	1.4050(4)
260.6	1.4009(5)
264.5	1.4014(4)
272.2	1.3977(5)
277.3	1.3939(7)
286.8	1.3898(5)
292.6	1.3848(6)
309.6	1.3778(5)

Supplementary Table S2. ***hP*4 *c/a* Axial Ratios to 310 GPa.** The c/a axial ratio of hexagonal *hP*4-Na, with uncertainties, as determined from Le Bail fits to diffraction profiles obtained between 206 and 310 GPa.

Reflection	I	σ_I	atomic-$hP4$ I_{calc}	pseudo-$hP4$ I_{calc}	electride-$hP4$ I_{calc}
$(0,0,2)$	74.4	54.6	0.0	119.4	97.9
$(1,\bar{1},0)$	67406.5	5502.7	90161.6	86801.3	83272.1
$(1,\bar{1},\bar{1})$	204765.5	16714.1	228399.9	224895.0	218892.3
$(1,0,2)$	365686.0	28596.4	424117.3	436140.1	434144.2
$(1,0,3)$	65142.5	4168.2	68436.2	69378.7	70516.0
$(1,0,4)$	13394.2	650.1	9390.4	9473.8	9587.2
$(1,0,5)$	11102.2	661.2	10606.7	10592.0	10578.5
$(2,\bar{1},4)$	108839.0	12584.2	81899.4	82124.0	82435.0
$(2,0,0)$	20041.6	2324.3	25721.5	26073.5	26480.5
$(2,0,1)$	71031.6	5807.1	67221.8	68147.9	69268.0
$(2,0,2)$	163491.5	13356.1	135774.8	137705.7	139716.7
$(2,0,3)$	35777.5	4158.9	24693.7	24880.6	25139.7
$(2,0,4)$	5123.4	671.0	3860.2	3858.5	3858.2
$(3,\bar{1},3)$	13519.1	1606.2	10331.5	10314.9	10297.5
$(3,\bar{1},4)$	2871.6	448.3	1772.7	1758.7	1740.1
$(4,\bar{1},0)$	820.1	183.1	1731.4	1717.4	1698.6
$(4,\bar{1},3)$	3086.7	469.2	2417.0	2385.0	2334.7
$(4,0,2)$	2193.0	368.4	5658.7	5575.1	5438.0

Supplementary Table S3. Observed and Calculated Intensities. Observed intensities and uncertainties (averaged over symmetry-equivalents) for each unique reflection and the calculated intensities from each model.

**Crystal Data**	
Chemical Formula	Na
M_r	22.99
Crystal System, space group	Hexagonal, P 6_3/m m c
Temperature (K)	293
Pressure (GPa)	223
a, b, c (Å)	2.903(5), 2.903(5), 4.139(2)
α, β, γ (°)	90, 90, 120
V (Å^3/atom)	30.21(11)
Z	4
Radiation Type	Synchrotron, $\lambda = 0.3738$Å
μ (mm^{-1})	0.291
Crystal Size	$0.001 \times 0.001 \times 0.001$
**Data Collection**	
Diffractometer	ESRF ID27, EIGER2 X CdTe 9M detector
No. of reflections: measured, independent, observed [$I > 3\sigma(I)$],	28, 18, 17
R_{int}	0.047
($\sin\theta_{max}/\lambda$) (Å^{-1})	0.835

Supplementary Table S4. Crystal data and data collection details for the single-crystal sample of $hP4$-Na at 223 GPa.

**Model**	atomic-$hP4$	pseudo-$hP4$	electride-$hP4$
**Refinement**			
$R_1(R)$	0.1053	0.1027	0.0972
$R_2(wR)$	0.1465	0.1409	0.1365
No. of reflections	18	18	18
No. of parameters	2	3	2
$\Delta\rho_{min}$, $\Delta\rho_{max}$ (eÅ^{-3})	$-1.25, 0.84$	$-1.21, 0.95$	$-1.16, 0.97$
**Crystal Structure**			
Na1 (x y z)	(0, 0, 0)	(0, 0, 0)	(0, 0, 0)
Na2 (x y z)	(1/3, 2/3, 3/4)	(1/3, 2/3, 3/4)	(1/3, 2/3, 3/4)
H^- (x y z)		(1/3, 2/3, 1/4)	
ICA (x y z)			(1/3, 2/3, 1/4)
$U_{iso,Na}$ (Å^2)	0.0223	0.0227	0.0232
U_{iso,H^-} (Å^2)		0.2121	
$U_{iso,ICA}$ (Å^2)			0

Supplementary Table S5. Refinement and crystal structure details for the different models of $hP4$-Na.

Supplementary References

[1] Brown, P.J., Fox, A.G., Maslen, E.N., O'Keefe, M.A., Willis, B.T.M.: Intensity of diffracted intensities. In: International Tables for Crystallography, pp. 554–595. International Union of Crystallography, Chester, England (2006). https://doi.org/10.1107/97809553602060000600

[2] Dovesi, R., Erba, A., Orlando, R., Zicovich-Wilson, C.M., Civalleri, B., Maschio, L., Rérat, M., Casassa, S., Baima, J., Salustro, S., Kirtman, B.: Quantum-mechanical condensed matter simulations with crystal. WIREs Computational Molecular Science **8**(4), 1360 (2018) https://doi.org/10.1002/wcms.1360

[3] Heyd, J., Scuseria, G.E.: Assessment and validation of a screened coulomb hybrid density functional. The Journal of Chemical Physics **120**, 7274–7280 (2004) https://doi.org/10.1063/1.1668634

[4] Heyd, J., Scuseria, G.E.: Efficient hybrid density functional calculations in solids: Assessment of the heyd–scuseria–ernzerhof screened coulomb hybrid functional. The Journal of Chemical Physics **121**, 1187–1192 (2004) https://doi.org/10.1063/1.1760074

[5] Heyd, J., Peralta, J.E., Scuseria, G.E., Martin, R.L.: Energy band gaps and lattice parameters evaluated with the heyd-scuseria-ernzerhof screened hybrid functional. The Journal of Chemical Physics **123** (2005) https://doi.org/10.1063/1.2085170

[6] Racioppi, S., Storm, C.V., McMahon, M.I., Zurek, E.: On the Electride Nature of Na-hP4. Angewandte Chemie International Edition **62**(48), 202310802 (2023) https://doi.org/10.1002/anie.202310802

[7] Holzapfel, W.B.: Equations of state for solids under strong compression. High Pressure Research **16**(2), 81–126 (1998) https://doi.org/10.1080/08957959808200283

[8] Dewaele, A., Loubeyre, P., Mezouar, M.: Equations of state of six metals above 94 GPa. Physical Review B **70**, 094112 (2004) https://doi.org/10.1103/PhysRevB.70.094112

[9] Storm, C.V., McHardy, J.D., Finnegan, S.E., Pace, E.J., Stevenson, M.G., Duff, M.J., MacLeod, S.G., McMahon, M.I.: Behavior of rubidium at over eightfold static compression. Physical Review B **103**, 224103 (2021) https://doi.org/10.1103/PhysRevB.103.224103

[10] Finnegan, S.E., Pace, E.J., Storm, C.V., McMahon, M.I., MacLeod, S.G., Liermann, H.-P., Glazyrin, K.: High-pressure structural systematics in samarium up to 222 GPa. Physical Review B **101**, 174109 (2020) https://doi.org/10.1103/PhysRevB.101.174109

[11] Finnegan, S.E., Storm, C.V., Pace, E.J., McMahon, M.I., MacLeod, S.G., Plekhanov, E., Bonini, N., Weber, C.: High-pressure structural systematics in neodymium up to 302 GPa. Physical Review B **103**, 134117 (2021) https://doi.org/10.1103/PhysRevB.103.134117

[12] Akaike, H.: A new look at the statistical model identification. IEEE Transactions on Automatic Control **19**(6), 716–723 (1974) https://doi.org/10.1109/TAC.1974.1100705

[13] Barrett, C.S.: X-ray study of the alkali metals at low temperatures. Acta Crystallographica **9**(8), 671–677 (1956) https://doi.org/10.1107/s0365110x56001790

[14] Lundegaard, L.F., Gregoryanz, E., McMahon, M.I., Guillaume, C., Loa, I., Nelmes, R.J.: Single-crystal studies of incommensurate Na to 1.5 Mbar. Physical Review B **79**, 064105 (2009) https://doi.org/10.1103/PhysRevB.79.064105
